# Mixed Conduction in A-Site Double-Perovskite Na_1+x_La_1-x_Zr_2_O_6-δ_ Proton Conductors

**DOI:** 10.3390/ma17215211

**Published:** 2024-10-25

**Authors:** Wenlong Huang, Zheng Gao, Ying Li, Yushi Ding, Jiayao Lu, Chunsheng Zhuang, Pengfei Yue, Wei Zhang

**Affiliations:** 1 School of Metallurgy, Northeastern University, Shenyang 110819, China; 2 Liaoning Key Laboratory for Metallurgical Sensor Materials and Technology, Northeastern University, Shenyang 110819, China; 3 Institute of Applied Physics, Henan Academy of Sciences, Zhengzhou 450008, China

**Keywords:** perovskite, proton conductor, transport properties, mixed conduction

## Abstract

Perovskite-type proton conductors exist in two structural forms, ABO_3_ and $A_{2}B^{\prime }B^{\prime \prime }O_{6}$. In this study, novel A-site double-perovskite proton conductors ($A^{\prime }A^{\prime \prime }B_{2}O_{6}$) were proposed. Na_1+x_La_1-x_Zr_2_O_6-δ_ (x = 0, 0.1, 0.2) perovskites were prepared by a solid-state reaction at 1200 °C. However, raising the sintering temperature to 1300 °C resulted in the Na to volatilize, converting the Na_1.1_La_0.9_Zr_2_O_6-δ_ into La_0.9_Zr_2_O_6-δ_. The conductivities of these materials in a humid atmosphere were tested using electrochemical impedance spectroscopy, and their carrier transport numbers were measured using the defect equilibria model and concentration cell method. Na_1.1_La_0.9_Zr_2_O_6-δ_ and Na_1.2_La_0.8_Zr_2_O_6-δ_ are predominantly proton conductors, with Na_1.1_La_0.9_Zr_2_O_6-δ_ exhibiting the highest proton transport number of 0.52 at 800 °C. In contrast, NaLaZr_2_O_6_ is predominantly an electronic conductor, while La_0.9_Zr_2_O_6-δ_ functions as an oxide ion conductor. Due to their high protonic transport numbers, these Na_1+x_La_1-x_Zr_2_O_6-δ_ A-site double-perovskite oxides present a promising avenue for the development of proton conductors.

## 1. Introduction

The pursuit of efficient energy conversion and storage technologies has catalyzed the exploration of advanced materials with unique properties. Perovskite-type materials have emerged as a promising class due to their versatile structural flexibility and potential for ionic conductivity. In particular, high-temperature proton conductors with perovskite structures have garnered significant attention due to their potential applications in fuel cells [1,2,3,4], electrochemical synthesis [5,6], hydrogen separation membranes [7,8], and electrochemical sensors [9,10]. These materials possess the chemical formula ABO_3_, where A typically represents a large cation and B denotes a smaller transition metal cation; their crystalline structures can be tailored to enhance protonic conductivity.

Among the ABO_3_-type proton conductors, Y- and Gd-doped BaCeO_3_ display the highest conductivities of more than 1.0 × 10^−2^ S·cm^−1^ at 600 °C in a humid, oxygen-containing atmosphere [11,12]. Meanwhile, In- and Sc-doped CaZrO_3_ shows the highest protonic transport numbers, with values maintained above 0.9 even at 700 °C [13,14]. The first $A_{2}B^{\prime }B^{\prime \prime }O_{6}$-type double perovskite was reported by Liang and Nowick in 1993 [15], and a similar $A_{3}B^{\prime }B_{2}^{\prime \prime }O_{9}$ double perovskite was reported by Nowick in 1995 [16]. The representative perovskite in this category is Ba_3_Ca_1.18_Nb_1.82_O_9-*δ*_ (BCN18), which has good overall performance in terms of conductivities and protonic transport numbers [16,17].

Fuel cells are electrochemical energy conversion devices that transform chemical fuels into electrical energy. A typical single proton conductor fuel cell (PCFC) primarily consists of three layers: the anode, the cathode, and the electrolyte. Due to the lower activation energy required for proton conduction compared to that of oxygen ion conduction, a typical PCFC can achieve efficient energy conversion at relatively lower temperatures than traditional oxygen ion-based solid-oxide fuel cells. Choi [18] utilized a BaZr_0.4_Ce_0.4_Y_0.1_Yb_0.1_O_3-δ_ proton conductor, which has an average grain size of approximately 10 μm, by controlling the chemical potential of the A-site cation as the electrolyte. The cell exhibits an exceptional peak power density of 1.90 W·cm^−2^ at 650 °C. Similar to the structure and principles of PCFCs, proton conductors can facilitate nitrogen reduction through the directional migration of protons, enabling ammonia synthesis under atmospheric pressure. Klinsrisuk [19] reported that a cell utilizing BaCe_0.5_Zr_0.3_Y_0.16_Zn_0.04_O_3-δ_ as the electrolyte achieved an ammonia synthesis efficiency of 4 × 10^−9^ mol·s^−1^·cm^−2^ at 450 °C. Protons generate a Nernst electromotive force (EMF) when diffusing through proton conductors, and the hydrogen content in the atmosphere can be determined by measuring this EMF. Hydrogen sensors based on this principle have been employed to measure the hydrogen content in aluminum and copper melts [20,21]. When an electromotive force is actively applied to a proton conductor, protons move directionally within the conductor, facilitating the separation of hydrogen from mixed gasses such as coke oven gas [22]. In these applications, the electrolytes are predominantly BaCeO_3_-based and CaZrO_3_-based due to their high conductivities and protonic transport numbers. Future research may benefit from focusing on enhancing these two properties to improve the performance of proton conductors in various applications.

Due to technological limitations, researchers find it challenging to directly observe the movement of protons. In early studies, charge carriers in compounds such as SrCeO_3_, KTaO_3_, and BaCeO_3_ were considered isolated protons [23,24,25]. However, protons are the only ions devoid of any electrons and possess a very high binding energy. In perovskites, oxygen ions carry a negative charge; thus, protons are bound to oxygen ions due to Coulomb forces. Cook [26] was the first to propose that protons in perovskites exist in the form of hydroxyl groups or hydroxide ions (${OH}_{O}^{\cdot }$). Münch [27] utilized quantum molecular dynamics to study protonic behavior in various perovskite proton conductors and suggested that there is a relationship between the amplitude of ions, the distance between adjacent oxygen ions, and proton migration. Münch proposed that protons rotate around oxygen ions through hydrogen bonding and jump to neighboring oxygen ions during lattice vibrations. Additionally, the generation of protons in proton conductors depends on catalysis by oxygen vacancies, as shown in Equation (1) [28]. These oxygen vacancies can also provide storage sites for protons [29].

However, oxygen vacancies also provide conductive pathways for oxygen ions, resulting in oxygen ionic conduction in proton conductors. Additionally, oxygen vacancies can catalyze the conversion of oxygen molecules into holes, leading to hole conduction, as shown in Equation (2). The conductivities of oxygen ions and holes in proton conductors can negatively impact performance in applications such as hydrogen sensors [30] and fuel cells [31]. Therefore, the concentration of oxygen vacancies needs to be adjusted to achieve proton conductors with balanced overall performance.

(1) $$H_{2}O+V_{O}^{\cdot \cdot }+O_{O}^{X}↔2{OH}_{O}^{\cdot }$$

(2) $$1/2O_{2}\left(g\right)+V_{O}^{\cdot \cdot }↔O_{O}^{X}+2h^{\cdot }$$

In ABO_3_-type proton conductors, the generation of oxygen vacancies is attributed to the doping of low-valence elements at the B-site, as shown in Equation (3).

(3) $$AO+\left(1-x\right)BO_{2}+0.5xM_{2}O_{3}=\;A_{A}+\left(1-x\right)B_{B}+xM_{B}^{\prime }+0.5xV_{O}^{\cdot \cdot }+(3-0.5x)O_{O}^{X}$$

Here, the oxidation states of A, B, and M are +2, +4, and +3, respectively, and M is typically a rare earth element.

In $A_{2}B^{\prime }B^{\prime \prime }O_{6}$-type proton conductors, oxygen vacancies can be generated by varying the concentrations of B′ and B″, as shown in Equation (4).

(4) $$2AO+\left(0.5+0.5x\right)B_{2}^{\prime }O_{3}+\left(0.5-0.5x\right)B_{2}^{\prime \prime }O_{5}=\;2A_{A}+{B^{\prime }}_{B^{\prime }}+x{B^{\prime }}_{B^{\prime \prime }}+\left(1-x\right){B^{\prime \prime }}_{B^{\prime \prime }}+xV_{O}^{\cdot \cdot }+(6-x)O_{O}^{X}$$

Here, the oxidation states of A, $B^{\prime }$, and $B^{\prime \prime }$ are +2, +3, and +5, respectively, and $B^{\prime \prime }$ is typically Nb or Ta. As demonstrated by this equation, the presence of additional doping elements is not required for the generation of oxygen vacancies in $A_{2}B^{\prime }B^{\prime \prime }O_{6}$-type proton conductors.

To the best of our knowledge, no studies on A-site double-perovskite proton conductors ($A^{\prime }A^{\prime \prime }B_{2}O_{6}$) have been reported to date. We speculate that oxygen vacancies can be created in these perovskite materials by adjusting the concentration ratio of A’ and $A^{\prime \prime }$, which would provide greater opportunities for customizing the structures of perovskite proton conductors. Additionally, these proton conductors would not require the incorporation of rare earth elements, Nb, Ta, or other costly materials, which would significantly reduce the overall material costs. In this study, Na_1+x_La_1-x_Zr_2_O_6-δ_ (x = 0, 0.1, 0.2) perovskites were fabricated using a solid-state reaction sintering process. The conductivities of these perovskite materials in atmospheres with various oxygen levels and water vapor partial pressures were measured at 500–800 °C via electrochemical impedance spectroscopy (EIS). The effects of atmosphere and temperature on the partial conductivities and transport numbers of protons, oxygen ions, and holes were systematically assessed. Furthermore, the impact of sintering temperature on Na volatilization, material composition, and conductivity was also investigated.

## 2. Experimental Details

### 2.1. Preparation and Characterization

Na_1+x_La_1-x_Zr_2_O_6-δ_ (x = 0, 0.1, 0.2) (NLZ0, NLZ1, and NLZ2) oxides were prepared through a solid-state reaction sintering process using Na_2_CO_3_ (Sinopharm; >99.8%), La_2_O_3_ (Macklin; >99.9%), and ZrO_2_ (Macklin; >99.9%). Briefly, stoichiometric mixtures of the chemical reagents were dispersed in ethanol and ball-milled for 10 h. Subsequently, the solvent was evaporated, and the dried powders were pressed into columns (*φ* 25 mm × *d* 15 mm) and calcined at 900 °C under air for 4 h to decompose the carbonates. The calcined powders were then pressed into disks (*φ* 10 mm × *d* 2 mm), and the disks were sintered at 1200 °C or 1300 °C in air for 4 h.

The ceramic disks were ground into powders and sieved through a 200-mesh screen for characterization. The crystallographic parameters of the sintered Na_1+x_La_1-x_Zr_2_O_6-δ_ powders were characterized by powder X-ray diffraction (PXRD) using Cu Kα radiation (40 kV and 30 mA) in the 2θ range of 5–90°. Inductively coupled plasma optical emission spectrometry (ICP-OES) and atom absorption spectrometry (AAS) were employed to verify the volatilization of sodium. A transmission electron microscope (TEM) was utilized to examine the microstructures of the final powders.

### 2.2. Conductivity Measurement

The conductivities of the prepared perovskites were determined by electrochemical impedance spectroscopy. Both sides of the sintered NLZ disks were coated with Pt paste, connected to Pt wires, and heated at 900 °C for 10 min to prepare porous Pt electrodes. The cell was installed in an airtight tube furnace equipped with a silicon carbide heater. Subsequently, different atmospheres containing various oxygen and water vapor partial pressures were flowed through the sample at a flow rate of 200 mL/min. The oxygen partial pressure was regulated using a mass flow controller (MFC) to mix O_2_, Ar, and 5%O_2_/Ar. A peristaltic pump was employed to inject water into the oxygen–argon mixed gas to control the water vapor pressure. AC impedance spectra were measured within the frequency range of 1–10^7^ Hz at a voltage of 500 mV. The impedance spectra were fitted using the ZSimpwin 3.10 software to obtain the resistance of each sample.

### 2.3. Transport Numbers

Oxides, particularly proton conductors, exhibit electronic conductivity only under low-oxygen partial pressures. However, oxides exhibit hole conductivity under typical air atmospheres. Therefore, in humid air, the prepared NLZ samples could be assumed to exhibit proton, oxide ion, and hole conductivity. The partial conductivities of proton, oxide ion, and hole were analyzed by a defect equilibria model [32,33]. The partial conductivities were calculated as follows:

(5) $$\frac{\sigma_{{OH}_{O}^{\cdot }}}{\sigma_{{OH}_{O}^{\cdot }}^{*}}=[{\left(1+\alpha /p_{H_{2}O}\right)}^{1/2}-1]p_{H_{2}O}/[{\left(1+\alpha \right)}^{1/2}-1]$$

(6) $$\frac{\sigma_{V_{O}^{\cdot \cdot }}}{\sigma_{V_{O}^{\cdot \cdot }}^{*}}={\left[{\left(1+\alpha /p_{H_{2}O}\right)}^{1/2}-1\right]}^{2}p_{H_{2}O}/\alpha$$

(7) $$\frac{\sigma_{h^{\cdot }}}{\sigma_{h^{\cdot }}^{*}}=[{\left(1+\alpha /p_{H_{2}O}\right)}^{1/2}-1]{{(p}_{H_{2}O}/\alpha )}^{1/2}p_{O_{2}}^{1/4}$$

where $\sigma_{{OH}_{O}^{\cdot }}^{*}$ represents the proton conductivity ($p_{H_{2}O}$ = 1 atm), $\sigma_{V_{O}^{\cdot \cdot }}^{*}$ refers to the oxide ion conductivity ($p_{O_{2}}$ = 1 atm), and $\sigma_{h^{\cdot }}^{*}$ corresponds to the hole conductivity ($p_{O_{2}}$ = 1 atm and $p_{H_{2}O}$ = 0). The parameter α can be calculated as in Equation (8):

(8) $$\alpha =\frac{8\times 0.1\times N_{A}}{V_{m}\times K_{H_{2}O}}$$

where 0.1 is the variation in the stoichiometric ratio *x* of Na to La in NLZs, N_A_ denotes Avogadro’s number, V_m_ is the molar volume, and $K_{H_{2}O}$ is the equilibrium constant of Equation (1).

The transport numbers were calculated by determining the ratio of partial conductivities to total conductivities. In our previous calculations, we observed that the defect equilibria model typically fits oxides dominated by proton conductivity. However, this model generally fails to provide an analytical solution for mixed conductors that do not exhibit proton conductivity. Therefore, the concentration cell method was also used to address the complex mixed conductivity in the NLZ samples. The concentration cell used a similar atmosphere to that employed in the defect equilibria model. The concentration cell can be represented as follows:

O_2_ concentration cell:

Ar + 10%O_2_
+ 2.34 kPaH_2_O, Pt | NLZs | Pt, Ar + 30%O_2_ + 2.34 kPaH_2_O

Water vapor concentration cell:

Ar + 20%O_2_
+ 0.61 kPaH_2_O, Pt | NLZs | Pt, Ar + 20%O_2_ + 4.25 kPaH_2_O

The mixed atmosphere was prepared as described in Section 2.2. The observed electromotive force (EMF) was recorded by a Sourcemeter (Keithley 2450, Cleveland, OH, USA). Theoretical EMF values were calculated by the Nernst equation [34]:

(9) $$E_{th}=\frac{RT}{4F}\left(t_{O^{2-}}+t_{{OH}_{O}^{\cdot }}\right)\ln\left(\frac{pO_{2}^{\prime }}{pO_{2}^{\prime \prime }}\right)-\frac{RT}{2F}t_{{OH}_{O}^{\cdot }}\ln\left(\frac{pH_{2}O^{\prime }}{pH_{2}O^{\prime \prime }}\right).$$

The transport numbers of ions *t*_ion_ (*E*_obs_/*E*th) and holes $t_{h^{\cdot }}$ (1 − *E*_obs_/*E*th) were obtained with the O_2_ concentration cell. The transport numbers of protons $t_{{OH}_{O}^{\cdot }}$ (−$E_{obs}^{\prime }/E_{th}^{\prime }$) and oxide ions $t_{O^{2-}}$ (*t*_ion_ − $t_{{OH}_{O}^{\cdot }}$) were obtained with the water vapor concentration cell.

## 3. Results and Discussion

### 3.1. Phase Composition and Structural Analysis

**Figure 1:**
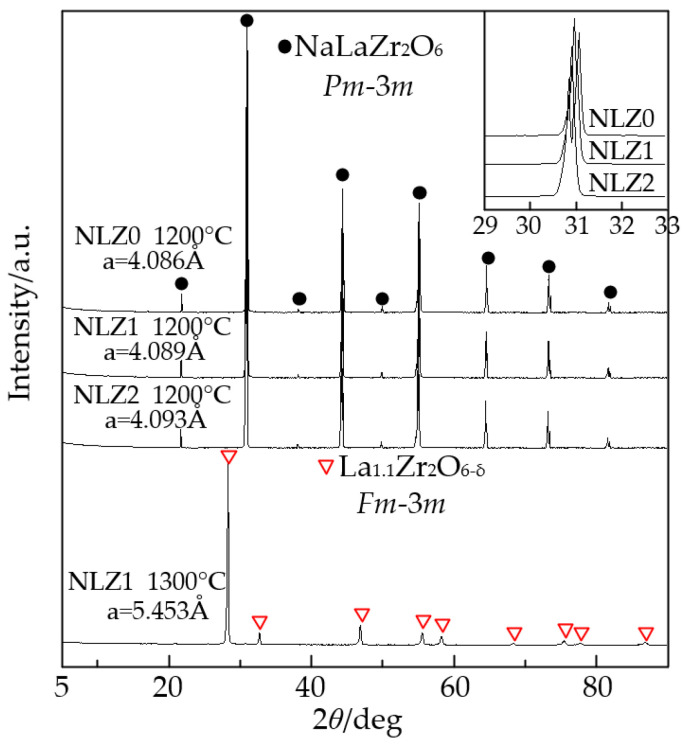
PXRD patterns of NLZ powders sintered at 1200 °C and 1300 °C.

A relatively short sintering time of 4 h was employed to prepare the NLZ samples in this study compared to the conventional sintering time of 10 h for proton conductors. This approach was adopted to control the sodium content, as alkali metal elements tend to volatilize at high temperatures. The results from ICP-OES and AAS indicated that the stoichiometric ratios of NLZ0, NLZ1, and NLZ2 were 0.999:1.000:2.000, 1.099:0.899:2.000, and 1.198:0.799:2.000, respectively, demonstrating that sodium did not volatilize after calcination at 900 °C. However, the stoichiometric ratios of NLZ0, NLZ1, and NLZ2 after sintering at 1200 °C for 4 h were found to be 0.905:1.000:2.000, 0.995:0.899:2.000, and 1.087:0.799:2.000, respectively, indicating that sintering led to the volatilization of approximately 10% of the sodium. Therefore, the sodium content was increased by 10% during sample preparation to ensure that the desired stoichiometric ratios were achieved. Sintering at 1300 °C for 4 h resulted in the uncontrollable volatilization of all sodium, rendering it impossible to maintain the stoichiometric ratio through the addition of extra sodium. The PXRD patterns of the NLZs sintered at 1200 °C, and 1300 °C in air for 4 h are shown in Figure 1. The crystallite sizes of the NLZs were calculated using the Debye–Scherrer equation, resulting in a size range of 50–60 nm. The stoichiometric NLZs sintered at 1200 °C showed diffraction peaks ascribed to the *Pm*-3*m* phase, and the peaks of Na_2_O, La_2_O_3_, and ZrO_2_ were not observed. This confirmed that the NLZs were successfully synthesized with an additional 10% sodium. Noteworthily, the lattice of an ABO_3_ perovskite becomes distorted when the ionic radii of the A- and B-site elements differ significantly, leading to a transition from a cubic phase to a monoclinic phase. Generally, when the tolerance factor *t* of a perovskite deviates from 1, the perovskite tends to transform into the *Pnma* phase [35]. The *t* values of the NLZs ranged from 0.924 to 0.925, indicating a high degree of distortion. Nevertheless, a cubic *Pm*-3*m* phase was still observed. Therefore, we speculate that the distortion of the BO_6_ octahedra in these perovskites can be primarily attributed to the ionic radius of the B-site elements, while the ionic radius of the A-site elements has a relatively minor impact on the crystal structure. Figure 1 shows that the lattice parameter a and the cell volume of the NLZs increased with increasing sodium content. This is due to the fact that the ionic radius of Na^+^ (1.39 Å; coordination: 12) is larger than that of La^3+^ (1.36 Å; coordination: 12).

When the sintering temperature was raised to 1300 °C, all the sodium volatilized, and La_0.9_Zr_2_O_6-δ_ with an *Fm*-3*m* phase was obtained. La_0.9_Zr_2_O_6-δ_ has the same crystal structure as La_2_Zr_2_O_7_ [36], which is a pyrochlore-type proton conductor material. The chemical formula of La_0.9_Zr_2_O_6-δ_ indicates the presence of oxygen vacancies, which theoretically facilitate the catalysis of water molecules. However, the stoichiometry and oxygen vacancy concentration of La_0.9_Zr_2_O_6-δ_ significantly differ from the compound structure we aimed to create in this study, potentially leading to considerable differences in conductivity compared to our expectations. Therefore, in the subsequent analysis of carrier conductivity, the conductivity properties of both NLZs and La_0.9_Zr_2_O_6-δ_ were investigated.

### 3.2. Microstructure Characterization

**Figure 2:**
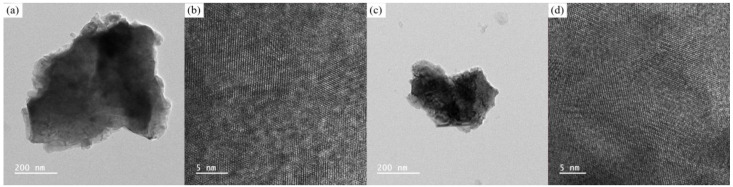
TEM images of NLZ1 sintered (**a**,**b**) at 1200 °C and (**c**,**d**) 1300 °C.

Figure 2a–d show the TEM images of the NLZ1 and La_0.9_Zr_2_O_6-δ_ powders, respectively. Significant grain boundary regions and amorphous phases were observed, indicating that the low sintering temperature resulted in inefficient sintering of the NLZs. Therefore, in this study, the conductive properties of the NLZs were mainly dominated by the conduction occurring at the grain boundaries.

### 3.3. Electrochemical Impedance Spectroscopy

**Figure 3:**
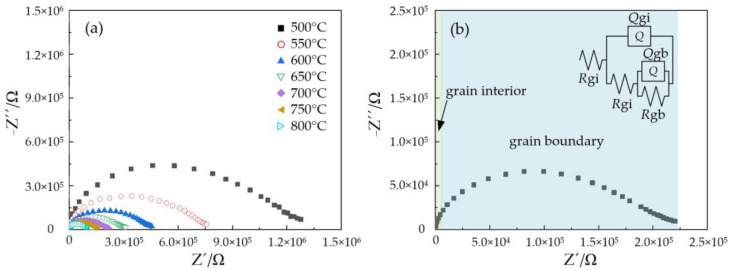
Nyquist plots of NLZ1 under an atmosphere of 99.9% O_2_ containing 2.34 kPa H_2_O (**a**) at 500–800 °C and (**b**) 700 °C.

The Nyquist plots of the NLZs measured in pure O_2_ containing 2.34 kPa H_2_O were obtained by electrochemical impedance spectroscopy, with those of NLZ1 exhibited in Figure 3. The resistance of NLZ1 decreased with increasing temperatures. Significant distortion was observed in the impedance semicircles, where the semicircles corresponding to the grain interior and grain boundary were mixed, making them difficult to distinguish. Therefore, the ZSimpwin 3.10 software was used to fit these Nyquist plots, and constant phase elements (*Q*) were used instead of capacitance (*C*). As shown in Figure 3b, these Nyquist plots were well fitted by the equivalent circuit R(Q(R(QR))). The resistances of the grain interior (*R*_gi_) and grain boundary (*R*_gb_) were obtained from the fitted data. Within the temperature range of 500–800 °C, all grain interior resistances were notably lower than the grain boundary resistances due to the low sintering temperature, which aligns with the microstructures described in Section 3.2. Iguchi [37] observed that dopant species slightly accumulate at the grain boundaries, where the equilibrium concentration of oxygen vacancies is higher compared to that in the grain interiors. Consequently, the NLZs contained a high concentration of oxygen ions and holes, which restricted the proton transport number. In addition, the conductivity under 2.34 kPa water vapor was lower than that under 0.61 kPa water vapor, indicating that a high water vapor pressure did not promote the hydration of the NLZs. This phenomenon has also been observed in other materials with large grain boundaries [38]. It can be inferred that the grain boundaries of the NLZs limited their hydration, potentially leading to a reduction in proton concentration (Equation (1)).

### 3.4. Conductivity and Transport Numbers of NLZs

**Figure 4:**
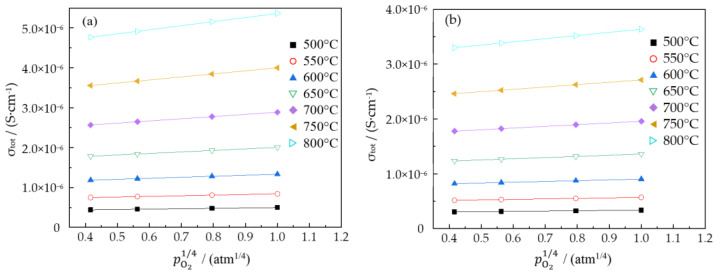
Conductivities of NLZ1 versus oxygen partial pressure under different water vapor partial pressures: (**a**) $p_{H_{2}O}$ = 0.61 kPa and (**b**) $p_{H_{2}O}$ = 2.34 kPa.

The changes in the total conductivities of NLZ1 versus $p_{O_{2}}^{1/4}$ under different water vapor partial pressures are provided in Figure 4. The total conductivities of NLZ1 linearly increased as a function of $p_{O_{2}}^{1/4}$, and this relationship was well fitted with Equation (2).

This relationship shows that total conduction in NLZ1 was dominated by hole conduction under the wet atmosphere. All the conductivities of the NLZs conformed to this relationship, illustrating the suitability of the defect equilibria model for describing the conductivity of these materials.

**Figure 5:**
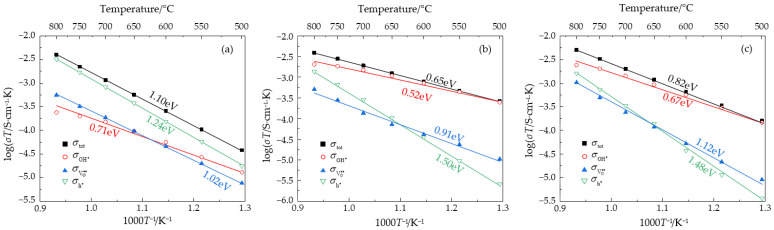
Arrhenius curves of total, proton, oxide ion, and hole conductivities of (**a**) NLZ0, (**b**) NLZ1, and (**c**) NLZ2.

To study the influence of stoichiometric ratios on the conductivities and transport numbers of the NLZs, the partial proton, oxide ion, and hole conductivities were calculated using the defect equilibria model. Moreover, a 21% O_2_ and 2.34 kPa H_2_O/Ar atmosphere was utilized to provide a comparison with other reported proton conductors, as listed in Table 1. Typical BaCeO_3_-based proton conductors (which exhibit the highest conductivities) and CaZr/HfO_3_-based proton conductors (which exhibit the highest transport numbers) are also listed in Table 1 for comparison. The proton, oxide ion, and hole activation energies of the NLZs were about 0.6, 1.0, and 1.4 eV, respectively. Proton conduction had a lower activation energy than the conduction of oxide ions and holes, in agreement with the activation energies reported in other studies. According to transition state theory, the activation energy is numerically approximately equal to the migration barrier of charge carriers, and the migration barrier is correlated with the migration distance of the charge carriers [39]. Therefore, these activation energies indicate that the prepared samples possess crystal structures similar to those of other typical proton conductors and can support the conduction of three types of charge carriers. Figure 5 shows the total conductivities and partial proton, oxide ion, and hole conductivities of the NLZs in wet air. Within the temperature range of 500–800 °C, NLZ0, NLZ1, and NLZ2 had total conductivities of 4.9 × 10^−8^ to 3.7 × 10^−6^, 3.4 × 10^−7^ to 3.6 × 10^−6^, and 2.1 × 10^−7^ to 4.7 × 10^−6^ S/cm, respectively.

These conductivities are significantly lower than those of typical proton conductors, indicating that the conduction of multiple carriers is not favored in the NLZs. Since conductivity is proportional to the product of the mobility and concentration of carriers, the activation energy data suggest that the migration of each carrier in the NLZs is comparable to that in doped CaZrO_3_. Therefore, it can be inferred that the concentration of each carrier in the NLZs is lower than that in typical proton conductors. This phenomenon can likely be attributed to the low sintering temperature of the NLZs, which results in the retention of a large number of grain boundaries. The conductive properties of the NLZs are primarily influenced by these grain boundaries, meaning that grain boundary irregularities restrict the concentration of carriers. The low hydration inferred from Figure 4 supports this hypothesis.

**Figure 6:**
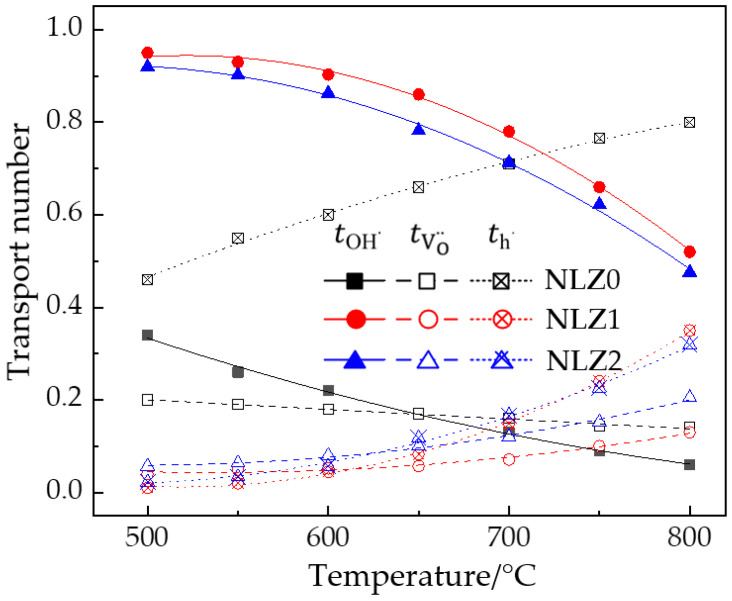
Transport numbers of proton, oxide ion, and hole carriers in NLZs.

Based on the Arrhenius curves shown in Figure 5, the transport numbers of the NLZs were calculated, as depicted in Figure 6. The protonic transport numbers of NLZ1 and NLZ2 were 0.95–0.52 and 0.92–0.48, respectively, indicating that the conductivities of these materials were dominated by protonic conduction in the temperature range of 500–800 °C. The protonic transport numbers of NLZ1 were higher than those of NLZ2. This phenomenon can be attributed to two reasons: (1) The unit cell volume of NLZ2 is larger than that of NLZ1, which necessitates that protons jump a longer distance in NLZ2. Consequently, NLZ2 has a higher proton migration barrier than NLZ1 [39]. (2) In typical proton conductors, optimal electrical conductivity is achieved when the theoretical oxygen vacancy concentration generated by doping is between 0.05/3 and 0.1/3 [1]. The theoretical oxygen vacancy concentrations of NLZ1(Na_0.55_La_0.45_ZrO_3-0.05_) and NLZ2(Na_0.6_La_0.4_ZrO_3-0.1_) are 0.05/3 and 0.1/3, respectively. An increase in the oxygen vacancy concentration may lead to the formation of defect clusters [43], which can reduce the effective oxygen vacancy concentration. On a microscopic level, protons in the form of hydroxyl groups can migrate to any of the eight surrounding oxygen atoms [28]. However, excessive oxygen vacancies can reduce the effective pathways available for proton jumping, which inhibits proton conduction.

Unlike NLZ1 and NLZ2, the conductivity of NLZ0 was dominated by hole conduction, which is consistent with studies on other proton conductors without oxygen vacancies [44]. The absence of oxygen vacancies in NLZ0 likely led to the following consequences: (1) water molecules were not easily catalyzed into protons (per Equation (1)); (2) in the absence of oxygen vacancy sites, the lattice did not provide sufficient proton storage sites [29]; and (3) adequate mobile pathways were not available for the migration of oxide ions. Therefore, NLZ0 exhibited extremely low ionic conductivities, and this material was primarily dominated by hole conduction. Overall, while the NLZs had considerably high protonic transport numbers, their overall conductivity remained low, indicating their lack of suitability for practical applications. However, these A-site double-perovskite oxides provide a new perspective for the further development of proton conductors.

### 3.5. Conductivity and Transport Numbers of NLZ1 Sintered at 1300 °C

**Figure 7:**
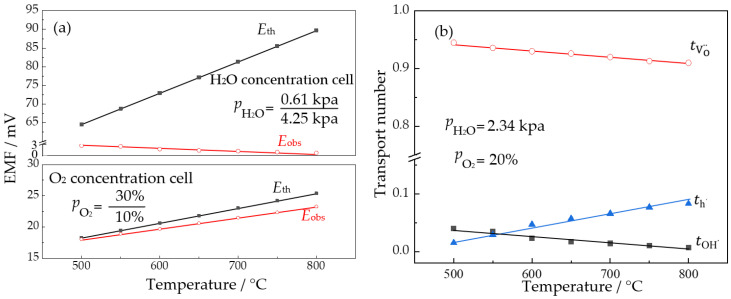
(**a**) EMF and (**b**) transport numbers of La_0.9_Zr_2_O_6-δ_ obtained using the concentration cell method.

To investigate the effect of sintering temperature on the NLZs, NLZ1 (which exhibited the highest proton transport number) was chosen for further study. The sintering of NLZ1 at 1300 °C led to the complete volatilization of Na, generating La_0.9_Zr_2_O_6-δ_. Nyquist plots of La_0.9_Zr_2_O_6-δ_ were obtained by EIS, and the conductivities of this material increased with the logarithm of oxygen partial pressure, resembling the trend shown in Figure 4. However, the defect equilibria model does not accurately describe low-proton-conducting materials, rendering analytical solutions difficult to obtain. Therefore, in this study, the vapor/oxygen concentration cell method was first used to measure the carrier transport numbers of La_0.9_Zr_2_O_6-δ_, and the partial conductivities of La_0.9_Zr_2_O_6-δ_ were calculated using the total conductivities and transport numbers. The electromotive force of the La_0.9_Zr_2_O_6-δ_ vapor/oxygen concentration cell is shown in Figure 7a, and the calculated carrier transport numbers are shown in Figure 7b.

The results from the oxygen concentration cell indicate that the electromotive force increased linearly with increasing temperature, indicating that ionic conduction is predominant in La_0.9_Zr_2_O_6-δ_. A low electromotive force was observed with the water vapor concentration cell, which was used to characterize the proton transport number. This implies that La_0.9_Zr_2_O_6-δ_ exhibits weak proton conduction. As shown in Figure 7b, La_0.9_Zr_2_O_6-δ_ had almost negligible proton transport numbers, and both the protons and oxygen ions decreased with increasing temperatures. Within the evaluated temperature range, oxygen ion conduction predominantly contributed to the conductivity of La_0.9_Zr_2_O_6-δ_, indicating that this material can be classified as an oxygen ion conductor.

**Figure 8:**
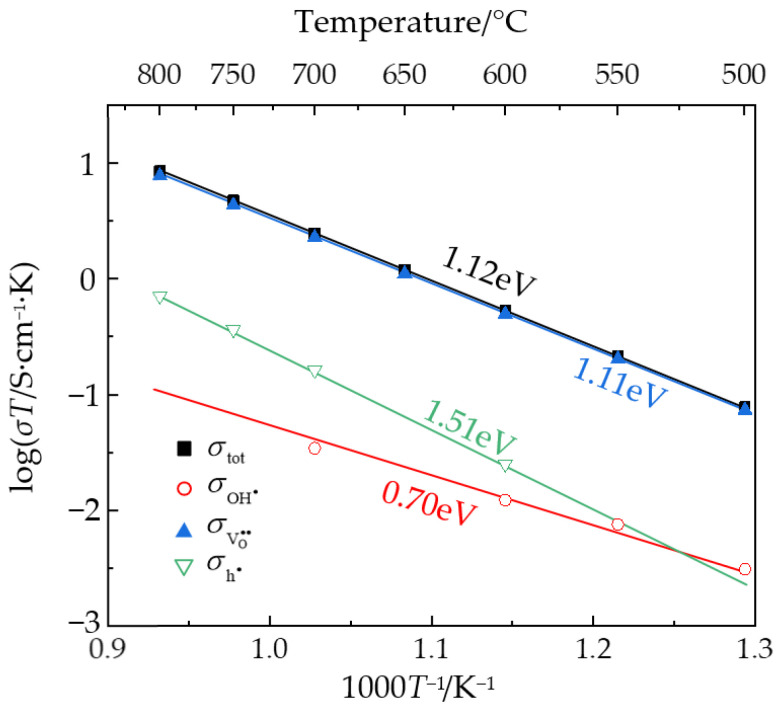
Arrhenius curves of total, proton, oxide ion, and hole conductivities of La_0.9_Zr_2_O_6-δ_.

The total conductivities of La_0.9_Zr_2_O_6-δ_ were calculated by fitting the impedance spectra. The partial conductivities of each carrier in wet air were calculated by multiplying the total conductivities and transport numbers, and Arrhenius curves of the conductivities are shown in Figure 8. Due to the relatively low electromotive force of protons and electrons in the concentration cell, the calculated conductivities exhibited significant error. Therefore, some of the partial conductivities shown in Figure 8 are based on estimated values. Within the temperature range of 500–800 °C, the total conductivities of La_0.9_Zr_2_O_6-δ_ were 1.0 × 10^−4^ to 7.9 × 10^−3^ S/cm. These values were three orders of magnitude higher than those of the NLZs sintered at 1200 °C and slightly lower than those of the BaCeO_3_-based proton conductors listed in Table 1, as well as typical ZrO_2_, CeO_2_-series oxygen ion conductors [40,41,42,45]. The activation energies of protons, oxide ions, and holes in La_0.9_Zr_2_O_6-δ_ were approximately 0.7, 1.1, and 1.5 eV, respectively.

Finally, crystal visualization software Vesta 3.4.7 was utilized to measure the distance between adjacent oxygen sites. The O-O distances in both the NLZs and La_0.9_Zr_2_O_6-δ_ were determined to be approximately 2.7 Å. Therefore, the activation energies for the carriers in La_0.9_Zr_2_O_6-δ_ were comparable to those in the NLZs. According to the PXRD analysis in this study, the crystal structure of La_0.9_Zr_2_O_6-δ_ is consistent with that of La_2_Zr_2_O_7_. The *Fm*-3*m* structure of La_0.9_Zr_2_O_6-δ_ contains a substantial number of cation and oxygen vacancies, and the excessive oxygen vacancy content of this material limits its conductivity.

## 4. Conclusions

Novel A-site double-perovskite proton conductors ($A^{\prime }A^{\prime \prime }B_{2}O_{6}$) were prepared in this study. Na_1+x_La_1-x_Zr_2_O_6-δ_ (x = 0, 0.1, 0.2) with the *Pm*-3*m* phase was prepared with 10% excess Na by a solid-state reaction process at 1200 °C for 4 h in air. However, raising the sintering temperature to 1300 °C caused the complete volatilization of Na, transforming Na_1.1_La_0.9_Zr_2_O_6-δ_ into *Fm*-3*m*-phase La_0.9_Zr_2_O_6-δ_. The total conductivities of Na_1+x_La_1-x_Zr_2_O_6-δ_ (x = 0, 0.1, 0.2) and La_0.9_Zr_2_O_6-δ_ under wet air at 500–800 °C were estimated to be 4.9 × 10^−8^–3.7 × 10^−6^, 3.4 × 10^−7^–3.6 × 10^−6^, 2.1 × 10^−7^–4.7 × 10^−6^, and 1.0 × 10^−4^–7.9 × 10^−3^ S/cm, respectively. Among the Na_1+x_La_1-x_Zr_2_O_6-δ_ (x = 0, 0.1, 0.2) proton conductors, Na_1.1_La_0.9_Zr_2_O_6-δ_ exhibited the highest proton transport number of 0.52 at 800 °C. In comparison, La_0.9_Zr_2_O_6-δ_ functioned primarily as an oxide ion conductor. The prepared materials had activation energies for proton, oxide ion, and hole of 0.6, 1.1, and 1.4 eV, respectively. Overall, the prepared Na_1+x_La_1-x_Zr_2_O_6-δ_ materials had high protonic transport numbers, indicating that these A-site double-perovskite oxides offer a promising avenue for the development of proton conductors.

## Figures and Tables

**Table 1:** Conduction data of typical proton conductors.

Composition	*T* (°C)	Atmosphere	*σ*_tot_ (S/cm)	$E_{tot}$/eV	$E_{{\mathrm{OH}}^{\cdot }}$/eV	$E_{V_{O}^{\cdot \cdot }}$/eV	$E_{h^{\cdot }}$/eV	$t_{{\mathrm{OH}}^{\cdot }}$	$t_{V_{O}^{\cdot \cdot }}$	$t_{h^{\cdot }}$	Ref.
BaCe_0.9_Y_0.1_O_3-δ_	600	∼0.1 kPa H_2_O, O_2_	~1 × 10^−2^	0.37	0.42	1.71	1.75	∼0.40	∼0.55	∼0.05	[40]
BaCe_0.85_Y_0.15_O_3-δ_	800	2.63 kPa H_2_O, Air	~4.9 × 10^−2^	0.69	0.29	0.92	1.27	∼0.33	∼0.22	∼0.45	[41]
BaCe_0.8_Y_0.2_O_3-α_	800	∼0.62 kPa H_2_O, O_2_	8.9 × 10^−3^	^−^	0.28	1.20	0.99	0.19	0.42	0.40	[42]
CaZr_0.9_Sc_0.1_O_3-α_	800	2.34 kPa H_2_O, Air	1.6 × 10^−3^	0.73	0.57	1.13	0.39	0.41	0.16	0.43	[14]
CaHf_0.9_Sc_0.1_O_3-α_	800	2.34 kPa H_2_O, Air	9.7 × 10^−4^	0.90	0.77	1.26	1.52	0.44	0.16	0.40	[14]
NaLaZr_2_O_6_	800	2.34 kPa H_2_O, Air	3.7 × 10^−6^	1.10	0.71	1.02	1.24	0.06	0.14	0.80	This work
Na_1.1_La_0.9_Zr_2_O_6-δ_	800	2.34 kPa H_2_O, Air	3.6 × 10^−6^	0.65	0.52	0.91	1.50	0.52	0.13	0.35	This work
Na_1.2_La_0.8_Zr_2_O_6-δ_	800	2.34 kPa H_2_O, Air	4.7 × 10^−6^	0.82	0.67	1.12	1.48	0.48	0.20	0.32	This work
La_0.9_Zr_2_O_6-δ_	800	2.34 kPa H_2_O, Air	7.9 × 10^−3^	1.11	0.70	1.11	1.51	0.01	0.91	0.08	This work

## Data Availability

All data generated or analyzed during this study are included in this published article.
